# Facile synthesis of hierarchically structured MIL-53(Al) with superior properties using an environmentally-friendly ultrasonic method for separating lead ions from aqueous solutions

**DOI:** 10.1038/s41598-022-06518-8

**Published:** 2022-02-16

**Authors:** Niusha Ahadi, Sima Askari, Amir Fouladitajar, Iman Akbari

**Affiliations:** Department of Chemical Engineering, Science and Research Branch, Islamic Azad University, Tehran, Iran

**Keywords:** Environmental sciences, Chemistry, Materials science

## Abstract

The present study aims at investigating sonochemically synthesized MIL-53(Al) and its applications in adsorption lead ions from aqueous solution. XRD, FESEM, BET, and FTIR analyses were employed to identify and characterize MIL-53(Al). The ultrasonic-assisted synthesis procedure results in reducing the synthesis time to 24 h; however, the conventional synthesis of MIL-53(Al) takes 3 days. Applying ultrasonic waves also leads to increase of the specific surface area up to 50% more than that of synthesized by the conventional method, as well as creating the hierarchical MIL-53(Al) structure which reduces the mass transfer limitation of ions into internal micropores. The optimum conditions for removing lead ions are pH of 6, Pb^+2^ ion concentration of 20 mg/L, contact time of 60 min, adsorbent dose of 0.04 g, and temperature of 318 K with the removal efficiency of 97.63%. The experimental adsorption equilibrium and kinetic data fit the Langmuir isotherm and pseudo-second-order kinetic models, respectively. Moreover, the usage of sonochemically synthesized MIL-53(Al), for the first time as an adsorbent in heavy metal removal points to the great potential of this new environmentally-friendly adsorbent in removing lead ions from aqueous solutions

## Introduction

Due to the fast development of trade and expansion of urbanization over the last few decades, pollution of surface and groundwater with heavy metals and their adverse effects on the health of living organisms as well as the consequent problems, have received considerable attention^1^. Among all heavy metals, lead is one of the most toxic elements. Lead is used as a significant raw material utilized in many industrial production operations such as batteries, radiation devices, solar cells, ceramics, and paint industries. Lead can cause nervous disorders such as IQ decline. In addition, it causes anemia and further side effects such as autoimmunity^2^, headache, insomnia, liver and kidney problems, gastrointestinal, and nervous system disorders, to name a few^3,4^. The World Health Organization established a 0.01 mg/L limit for lead ions in drinking water, but this value is no longer health-based and has been labeled provisional^5^. Therefore, Pb (II) decontamination of the contaminated water before being discharged into the water reservoir from industrial plants is of high significance.

A number of techniques such as adsorption, chemical precipitation, coagulation, membrane filtration, ion exchange and electro-reduction are applied to remove heavy metals from wastewater. Compared to other techniques in water treatment, adsorption is a more appropriate option because of its simplicity, efficiency, and economy^6,7^.

Different adsorbents have been developed to remove dissolved materials from the solution using ion adsorption mechanisms. In this regard, preparing a promising adsorbent is considered to be a significant challenge. Nano-porous compounds such as Metal–Organic Frameworks (MOFs) have received significant attention in recent years due to their vast contact surface areas, solvent stabilities, high porosity, well-organized pores, and regular particle size^8–10^. Further, MIL-53 is a group of MOFs produced by the Lavoisier Institute, which is a white powder with the chemical formula of [Al (OH) [(O_2_C)–C_6_H_4_–(CO_2_)]. Owing to the unique surface area, porosity, pores shape, size tunability, and adsorptive nature, MIL-53(Al), among the MILs family, can efficiently stimulate the scientific society^11,12^. MOFs are used in different fields such as catalysis, separation, gas storage, and drug delivery. Few studies have been conducted on their applications as the adsorbents used for removing hazardous substances such as volatile organic compounds and dyes from aqueous solutions^13–15^. The adsorption performance mainly depends on several characteristics of adsorbents such as the crystallinity, particle size, specific surface area and availability of the functional groups. In 2018 the AMCA-MIL-53(Al) was successfully developed by Alqadami et al., and its potential for eliminating lead(II) from aqueous solutions was investigated. The mentioned MOF has a low specific surface area but a high adsorption capacity of 390 mg/g, showing that its adsorption was mostly governed by functional groups^13^.

In the present study, MIL-53(Al) is synthesized through an environmentally-friendly method using ultrasonic waves and applied for the first time as an adsorbent in heavy metal removal. It is characterized using XRD, FESEM, BET, and FTIR analyses. The effects of solution temperature, pH, and adsorption dosage on removal efficiency as well as the kinetic and isotherm approaches are reported. The usage of ultrasound as a driving force lowers the reaction time and temperature which leads to decreasing the synthesis energy consumption dramatically. It enhances the adsorbent's properties such as surface area, which can boost the adsorbent features in removing lead ions from aqueous solutions. Moreover, ultrasound waves create the hierarchical structure which reduces the mass transfer limitation of ions into internal microspores.

## Materials and methods

### Materials and reagents

The primary materials used in the synthesis and adsorption tests were of analytical reagent grade. Aluminum (III) nitrate nonahydrate (Al–(NO_3_)_3_·9H_2_O) and 1,4-BenzeneDiCarboxylic (BDC) acid were used as Al(metal) sources and organic ligands, respectively, which were purchased from Merck, Germany. In addition, *N*,*N*-dimethylformamide (DMF), methanol (CH_3_OH), Hydrochloric acid (HCl), and sodium hydroxide (NaOH) were acquired. Moreover, the source of lead ions was [Pb(NO_3_)_2_] procured from Merck, Germany. Deionized (D.I.) water was also used to prepare all solutions.

### Preparation of hierarchically structured MIL-53(Al)

The present study employed a new environmentally-friendly method for ultrasonic-assisted synthesis (UAS) of MIL-53(Al). According to this method, (6.5 g) Aluminum (III) nitrate nonahydrate (Al-(NO_3_)_3_·9H_2_O) (98% purity) and (1.44 g) 1,4-BenzeneDiCarboxylic (BDC) acid (98% purity) with 25 ml of D.I water were stirred for 10 min at room temperature until complete dissolution is achieved. The mixture was then exposed to ultrasonic waves of 24 kHz and 100% intensity for 30 min after dissolving and then, it was transferred to 100 ml autoclave with black Teflon liner steel. Finally, an oven was used to heat the sample to 220 °C for 24 h. The autoclave was cooled down and the solid phase was separated using a centrifuge, and 35 ml of DMF solution (solid solvent) was added to remove unreacted terephthalic acids trapped in the pores of the samples. The autoclave was put back in the oven to be heated to 150 °C for 12 h. Finally, the solid phase was separated by centrifuge and washed with methanol; then, 40 ml of methanol was added to the sample, and the sample was dried in an oven at 150 °C overnight. The result is a solid white powder subjected to different analyses used for identifying and ensuring the correct formation of MIL-53(Al). In the end, the synthesized crystals were calcined at 650 K in the air for 5 h. In order to synthesis MIL-53(Al) with the conventional (CS) method, the primary mixture, including water, BDC, and Aluminum(III) nitrate nonahydrate with the same amount as ultrasonic synthesis, was stirred for 1 h at room temperature until complete dissolving. It then transferred to 100 ml autoclave with black Teflon liner steel and used the oven to heat the sample to 220 °C for 72 h. Finally, the autoclave was let to be cooled down. All the other experimental conditions took place the same as the ultrasonic synthesis method.

### Characterization

The morphology and composition characteristics of MIL-53(Al) were evaluated using Field Emission Scanning Electron Microscopy (FESEM) and a Seron AIS2100. In addition, X-Ray Diffraction (XRD) was employed to examine the crystalline structure recorded by the PW1730 diffractometer with λCuKα = 1.54056 A˚ and a step size of 0.05 at 40 kV and 30 mA. The BELSORP MINI ll instrument measured the Brunauer–Emmett–Teller (BET) analysis within the nitrogen adsorption–desorption isotherm method at (77 K) in order to detect structural properties of MIL-53(Al). Fourier-Transformed Infrared spectroscopy (FT-IR) was obtained using the PERKIN EKMER instrument at a resolution of 400–4000 cm^−1^. Energy-dispersive X-ray analysis (EDX) was used to determine the elemental composition of the UAS MIL-53(Al) samples. The analysis was performed using a TECSCAN MIRA ll field emission scanning electron microscope equipped with a SAMX detector (France). Further, AAS was used to measure the lead concentration using Model 3110 Perkin Elmer Atomic Absorption Spectrometer. The pH meter model, combined with a pH electrode, was of the 827 pH Lab (Metrohm, Swiss).

### Preparation of lead solutions

Millipore/D.I. water and lead (II) nitrate were used to prepare the lead (II) stock solution with a concentration of 1000 mg/L. Other lead (II) solutions with different concentrations were freshly prepared for each experiment using the stock solution.

### Evaluation of adsorbents performance

MIL-53(Al) adsorption capacities for Pb(ll) were presented at different dosages with known initial concentrations for fixed values of time, temperature, and pH. When the solution reached equilibrium, two solid and liquid phases were divided through filtration and then, Atomic Absorption Spectrometer (AAS) was employed to determine the remaining concentration of the Pb^+2^ ions in the solution. The pH of point of zero charges (pHpzc) of MIL-53(Al) was calculated by the method proposed by Faria et al.^16^. The effect of initial pH on lead ion adsorption was also examined at different pHs ranging from 2 to 7. The pH of the solution was fixed by adjusting HCl and NaOH 0.1 and 0.01 M to the solutions. Then, 0.04 g of MIL-53(Al) was added to every 40 ml of solution, and the agitation samples were placed on an incubator shaker at a speed of 315 rpm for three hours at room temperature. Eventually, the final concentration of lead(II) ion was analyzed using AAS.

Further, Pb^+2^ adsorption isotherm and other practical parameters were studied in the following experimental conditions: the concentration of 20–130 (mg/L), time range of 5–180 min, and different temperatures of 288, 298, 308, and 318 K. The adsorbent dosage ranges from 0.001 to 0.04 g. The kinetics investigations for Pb(ll) removal were evaluated through the analysis of Pb^+2^ adsorption in different time periods. The adsorption efficiency% and adsorption capacity of Pb(II) at equilibrium q_e_(mg/g) were determined using Eq. (1) and Eq. (2), respectively^17^:

1 $$Adsorption\, efficiency \%=\frac{{C}_{0}-{C}_{e}}{{C}_{0}}\times 100$$

2 $${q}_{e}(mg/g)=({C}_{0}-{C}_{e})\times \frac{V}{m}$$

where V(ml) is the volume of lead(II) ion solution; m(g) is the amount of MIL-53(Al); and C_0_ and C_e_ are the initial and equilibrium concentrations (mg/L) of lead(II) ion adsorbed, respectively.

## Result and discussion

### MIL-53(Al) characterization

**Figure 1:**
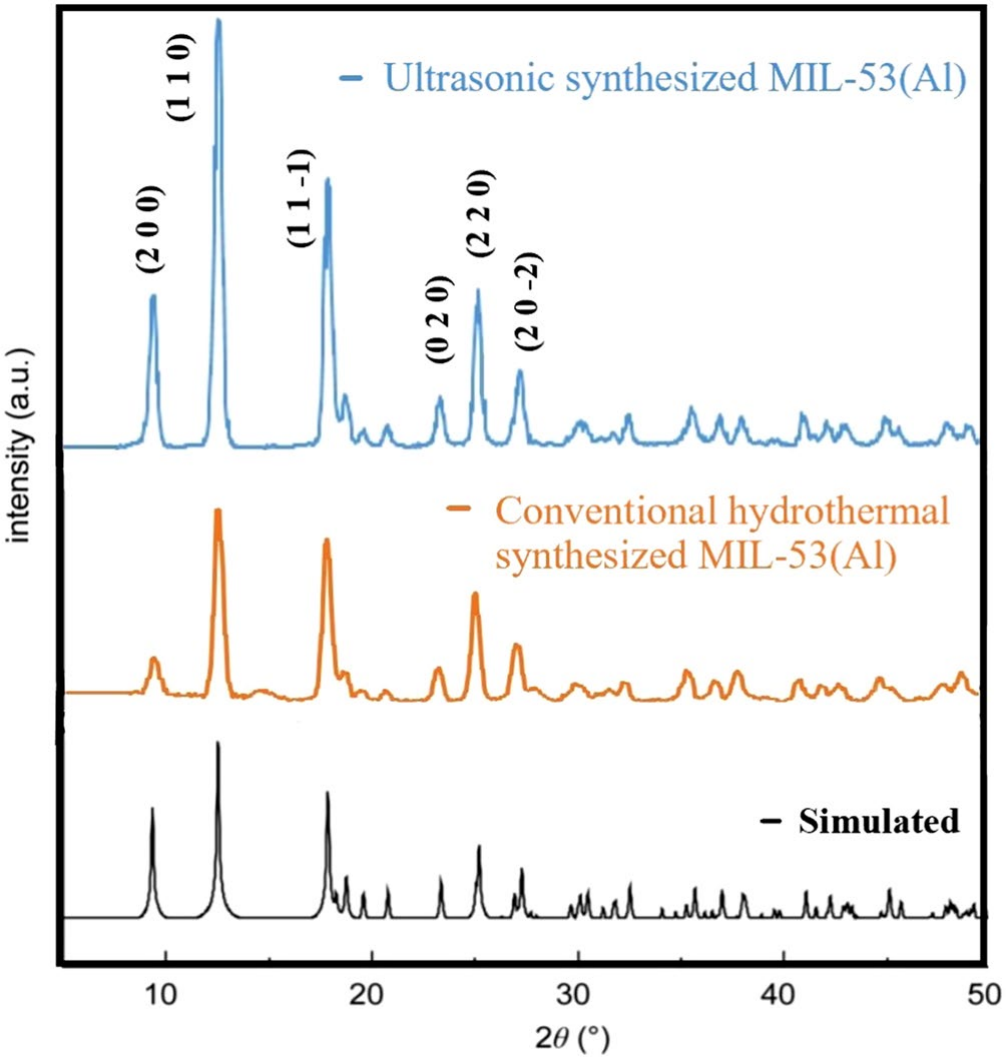
X-ray diffractograph of the ultrasonic and the conventional synthesized MIL-53(Al).

The XRD pattern of the synthesized MIL-53(Al) is presented in Fig. 1. These patterns are very similar to^18^. The XRD pattern of the UAS and CS MIL-53(Al) in this study shows the prominent characteristic peaks at 2θ = 9.38°, 12.58°, 17.93°, 23° 48, 25.28°, 27.33°, which match the peaks reported in^18^ and (CCDC file no. 220477), indicating that MIL-53(Al) is well crystallized and synthesized. Also, the difference in peak intensities is due to higher ultrasonic energy as a driving force, increasing nucleation rate and leading to a considerable amount of cavitation bubbles^19^.

**Figure 2:**
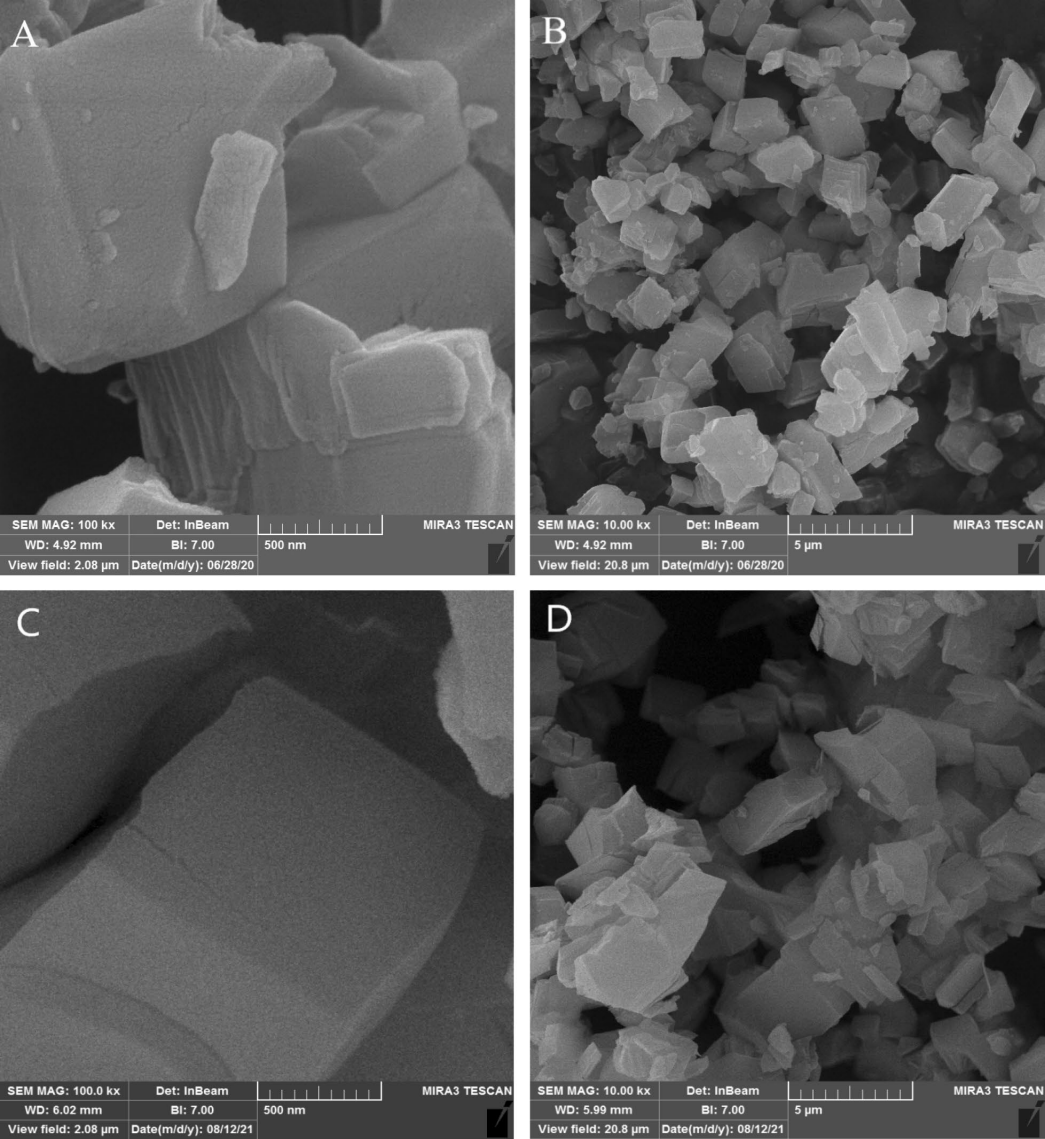
FESEM images of (**A**, **B**) UAS MIL-53(Al); (**C**, **D**) CS MIL-53(Al) at different magnifications.

The morphology of UAS and CS MIL-53(Al) samples with different magnifications were examined using FESEM, the results of which are presented in Fig. 2. According to this figure, the samples, which were synthesized through the sonochemical method, have octahedral shapes with crystal-like structures. Such structures are not aggregated; instead, they have a more uniform particle size which is smaller than the sample obtained by the conventional hydrothermal method. According to the standard conditions required for MOF crystal growth, faster nucleation rates result in smaller crystal sizes^20^. Following the FESEM characterization results, the sonochemical synthesized MIL-53(Al) has smaller uniformly distributed nanoparticles mainly because of the accelerated nucleation and shorter synthesis time required for sonochemical synthesis.

**Figure 3:**
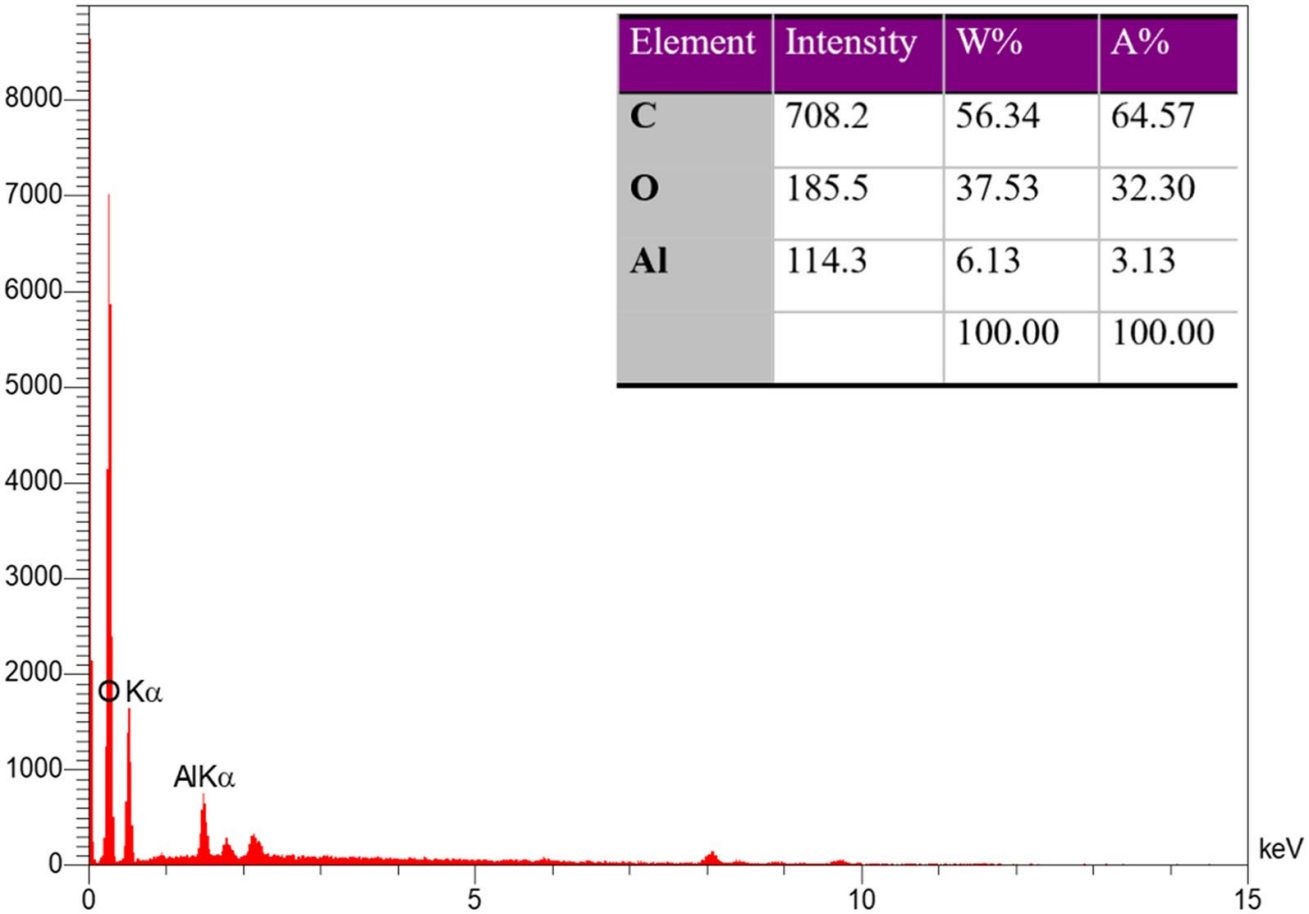
Elemental analysis plot and data.

The chemical composition of MIL-53(Al) was determined using energy-dispersive X-ray analysis (EDX).The presence of C(56.34%), O(37.53%), and Al(6.13%) in the Al-MOF is confirmed as demonstrated in Fig. 3.

**Figure 4:**
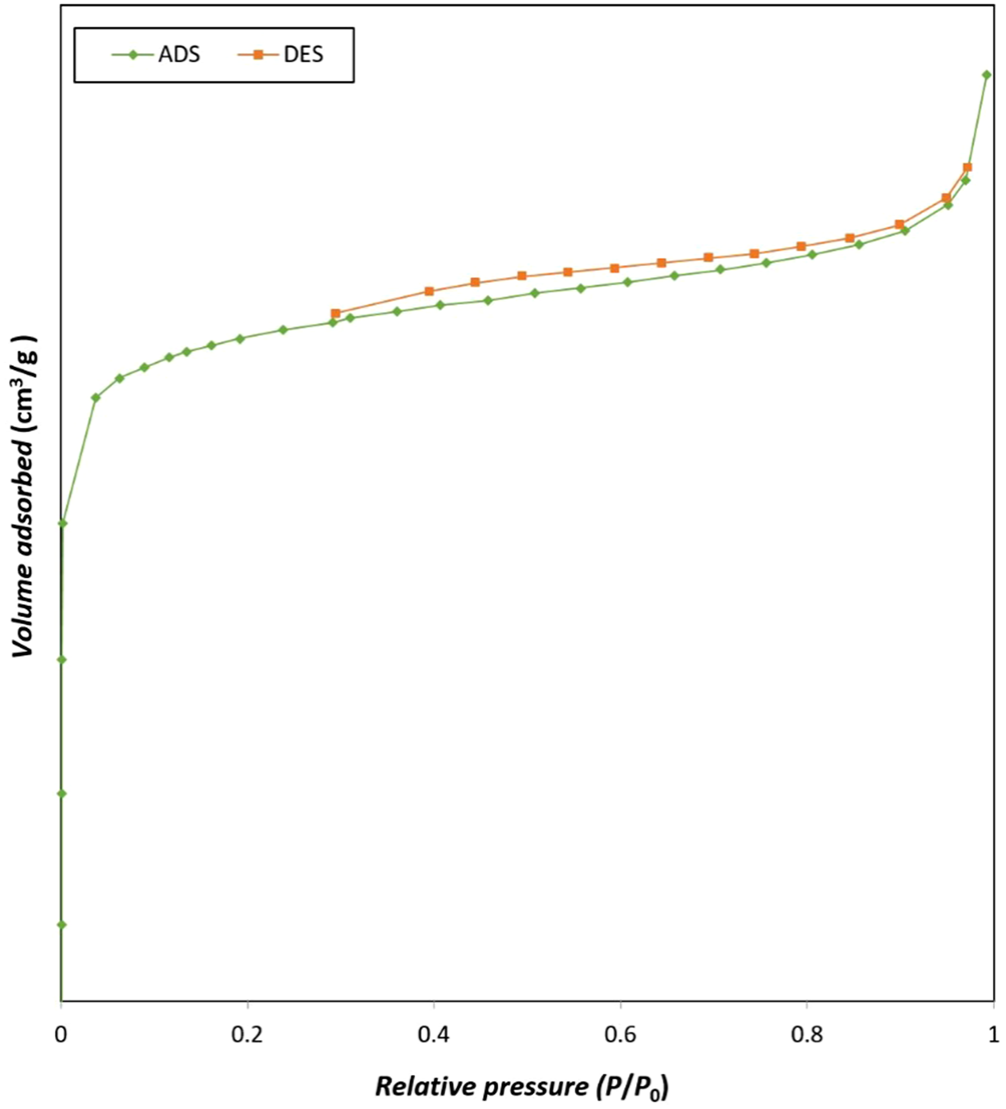
N_2_ adsorption–desorption isotherm of UAS MIL-53(Al).

The N_2_ adsorption–desorption isotherm of MIL-53(Al) is exhibited in Fig. 4. The synthesized sample resembles an IUPAC-type IV isotherm with a type H4 hysteresis loop, illustrating hierarchical structure including mesoporous and microporous in the sample while conventional synthesis MIL-53(Al) only possesses a microporous structure^21^. Furthermore, BET data of MIL-53(Al) are given in Table 1. The high crystallinity and porosity of the sample yield a high BET surface area of 1538.6 m^2^/g and a vast average pore size of 1.74 nm. BET surface area and average pore size are greater than those conventionally synthesized MIL-53(Al) introduced in the literature^21–23^; it could be explained by the effect of ultrasound waves on the crystalline structure of MIL-53(Al). Ultrasonic intensity as a driving force ends up in a substantial quantity of cavitation bubbles, and increases in crystallinity, leading to a higher BET expanse in line with the XRD pattern^19,24^. Moreover, the high intensity of ultrasonic waves creates mesopores in the structure of MOFs^25^, leading to a hierarchical ((meso- and micro-) pores) structures attending to a more significant average pore sizes^21,26^.

**Table 1:** Textural properties of different MIL-53(Al).

Sample	BET Surface area (m^2^/g)	Pore volume^a^ (cm^3^/g)	Average pore size (nm)	External surface area^b^ (m^2^/g)	Mesopore volume^c^ (cm^2^ /g)	Micropore volume^b^ (cm^2^/g)	Refs
UAS MIL-53(Al)	1538.6	0.671	1.7443	20.22	0.07	0.600	This study
CS MIL-53(Al)	1274.3	0.560	1.755	5.88	0.027	0.533	This study
MIL-53(Al)	1184	–	–	–	0	0.45	^21^
MIL-53(Al)	1073	0.46	–	–	–	–	^22^
MIL-53(Al)	1027	0.56	0.66	–	–	–	^23^

^a^Measured using t-plot method.

^b^Measured using t-plot method.

^c^Mesopore volume(From BJH model) = Totalpore volume – Micropore volume.

**Figure 5:**
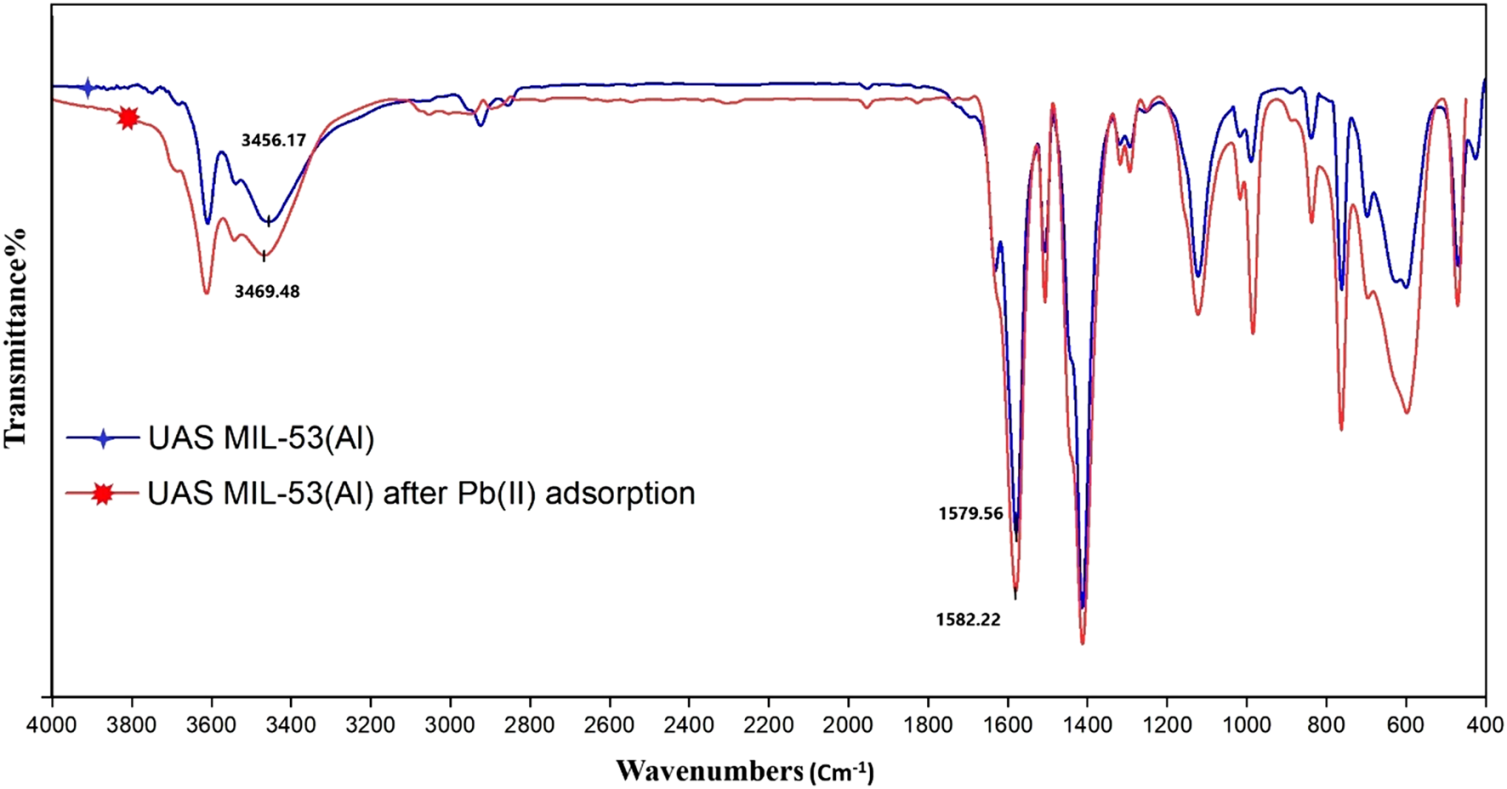
FT-IR spectrum of UAS MIL-53(Al) before and after Pb(II) adsorption.

Figure 5 illustrates the FT-IR spectrum of the synthesized UAS MIL-53(Al) which also shows the vibration bands in the wavelength range of 1700–1400 cm^−1^, indicating that the carboxylic functional groups are attached to aluminum. According to this figure, the firm observed peaks at 1507.87 and 1579.56 cm^−1^ belong to the (−COO) asymmetric stretching, and the characteristic peak at 1413.15 belongs to (−COO) symmetric stretching of the carboxyl vibration. No additional peak is observed at a wavenumber of 1700 cm^−1^, indicating that the free BDC acid molecules are completely removed from UAS MIL-53(Al) pores^18^. The peaks at 1631.9 cm^−1^ and vibration bands ranging from 3700 ± 3400 cm^−1^ confirm the observation of the bending and stretching modes of water as well as the signature of the hydroxyl group that links the aluminum particles^27^. The peak at 989.59 may relate to the bending vibrations of the hydroxyl group in the octahedral AlO_4_(OH)_2_ with trans corner-sharing^28^. The FTIR peaks of UAS MIL-53(Al) samples are in contant with those of referenced MIL-53(Al)^27^. Following the adsorption of Pb(II) ions, some bands in the FTIR spectra of UAS MIL-53(Al) shift to lower or higher wave numbers. For example, the bandwidth at 3456.17 cm^−1^ assigned to O–H moves to 3469.48 cm^−1^ revealing that stretching of the hydroxyl group was responsible for Pb(II) ion binding to the adsorbent. A minor shift in peak location is also noticed, moving from 1579.56 to 1582.22 cm^−1^ which could be related to the creation of co-ordinate bonds during the adsorption process^29^. The hydroxyl group band at 1631.9 cm^−1^ broadens after Pb(II) ions adsorption. Transformations in peak sizes and locations were seen in the UAS MIL-53(Al) spectra following adsorption of Pb (II). As illustrated in Fig. 5, it was discovered that the (−COO), (C–H), and (C–O–H) bends were also responsible for the efficient removal of Pb(II) ions.

## Adsorption studies

### UAS MIL-53(Al) adsorption performance in removing of different heavy metal ions

**Figure 6:**
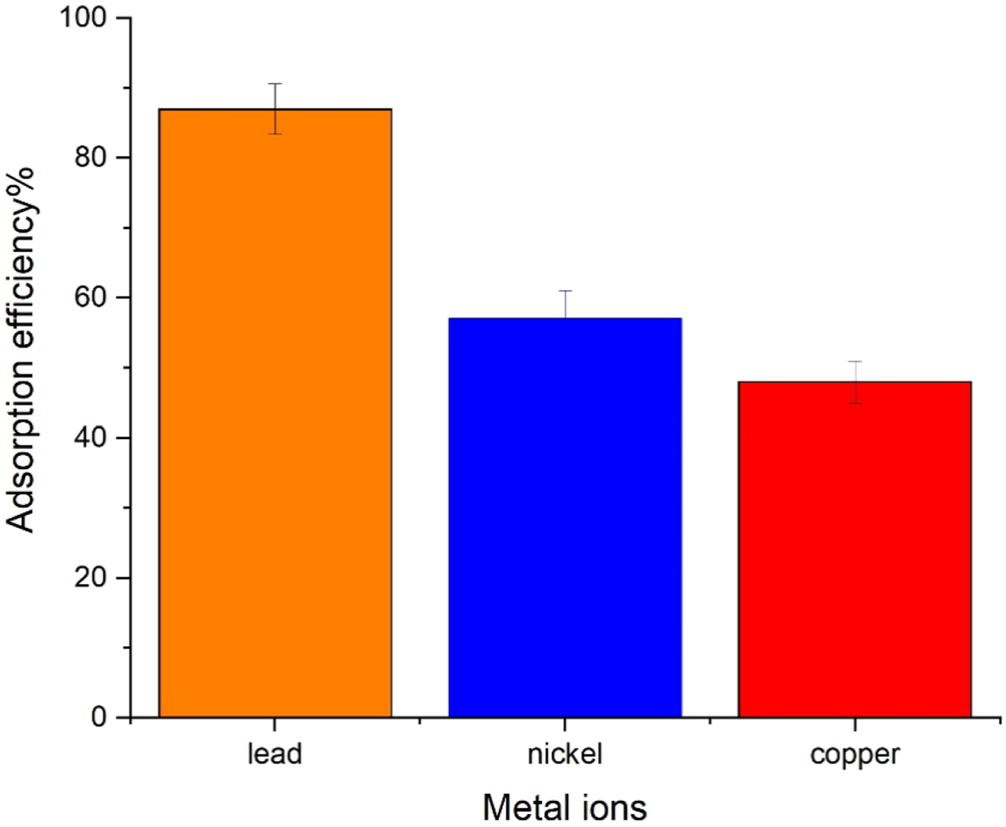
UAS MIL-53(Al) adsorption efficiency in removal of different heavy metal ions (20 ppm of intended heavy metal, 0.03 g UAS MIL-53(Al), and 40 ml solution, 298 K temperature).

In order to discover the selectivity of UAS MIL-53(Al) in removing the heavy metals from water, an adsorption experiment was carried out to measure the remaining concentration of these metals such as Pb^2+^, Ni^2+^, Cu^2+^ in the solution after adsorption. Three adsorption experiments, each containing 20 ppm of intended heavy metal, 0.03 g UAS MIL-53(Al), and 40 ml solution, were carried out to determine the selectivity of the adsorbent. According to Fig. 6, UAS MIL-53(Al) as an adsorbent can perfectly remove lead(II) from the solution since it comprises carbonyl groups. Therefore, the following tests were carried out using lead(II).

### Effect of pH on Pb(II) adsorption

**Figure 7:**
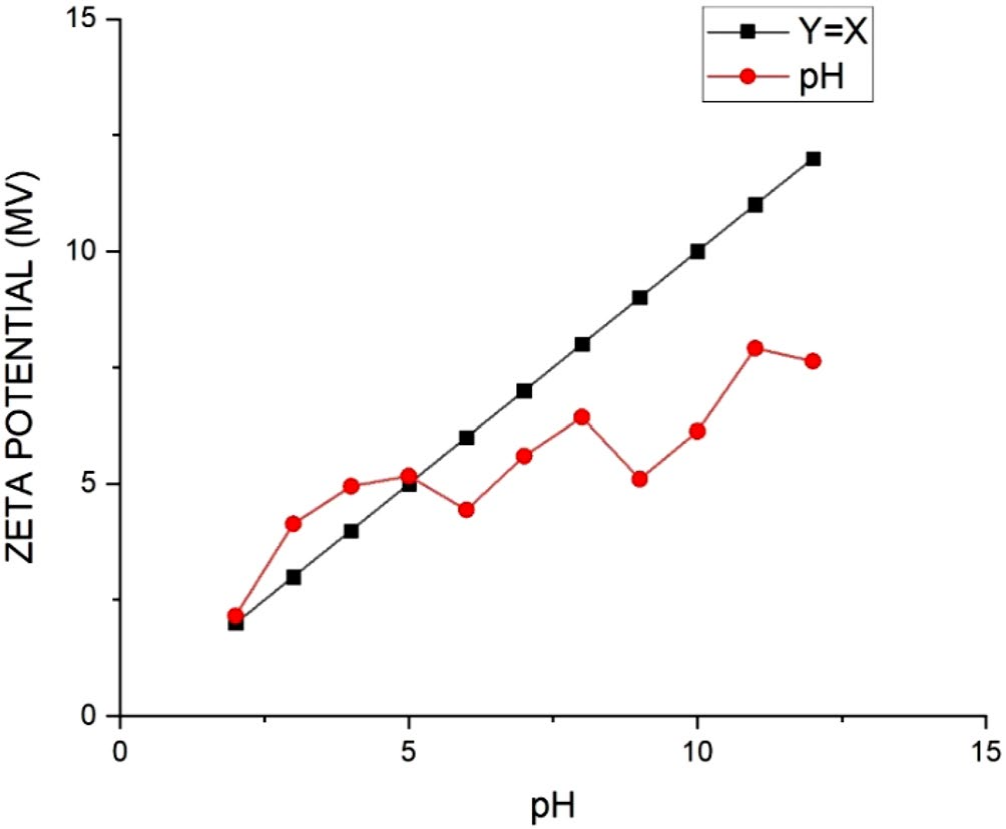
Effect of pH on the zeta potential of UAS MIL-53(Al) adsorbent.

**Figure 8:**
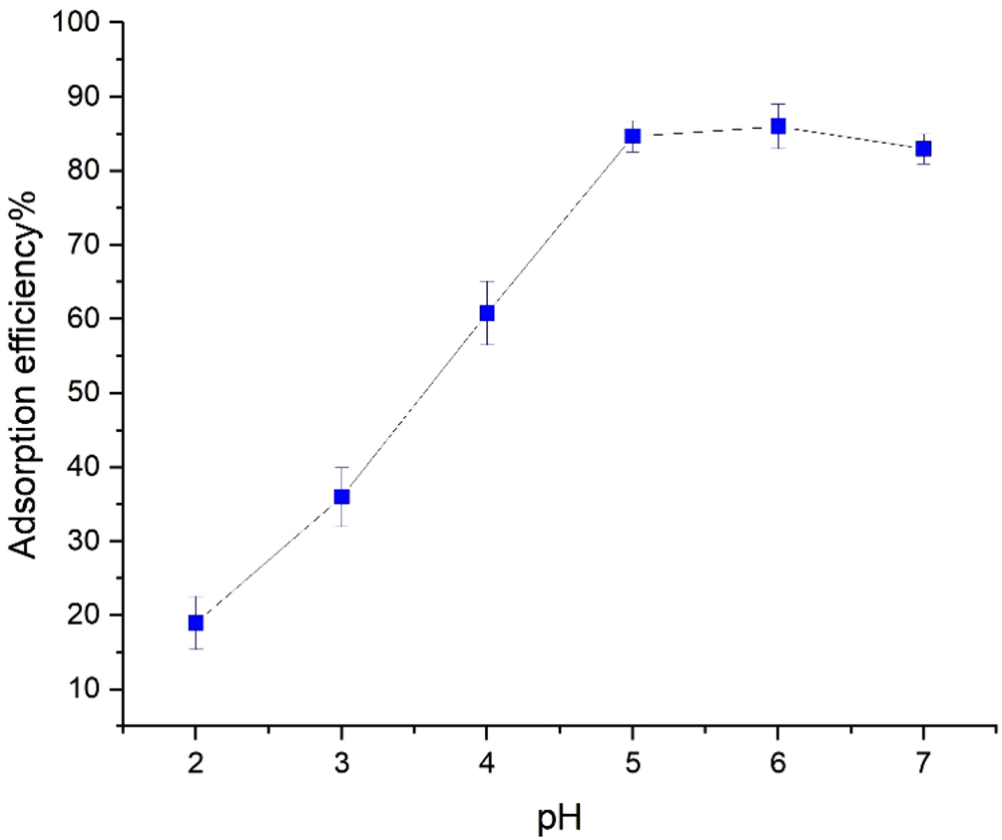
Effect of pH on Pb(II) adsorption (pH range (2–7), Pb(II) concentration (20 ppm), contact time (60 min), volume of the solution (40 ml), adsorbent dosage of 0.04 g, and temperature of 298 K).

The pH of the mixture is considered an essential parameter to be examined during the adsorption studies since changing pH can alter the surface charge of the adsorbent. Also, pH can strongly influence the solubility of metal ion^30^. First, in order to choose the pH range of the solution, the pH_pzc_ of the adsorbent should be analyzed. The pH_pzc_ of the solution which is the surface charge of the adsorbent is zero. In case pH_pzc_ > pH, the surface has a more positive charge by decreasing pH, and in case pH_pzc_ < pH, the surface has a more negative charge by increasing the pH. Figure 7 shows the pH_pzc_ in this study. The removal efficiency of Pb(II) ions were investigated in different initial pH ranges (2–7), as shown in Fig. 8, which were set using the required amount of HCl or NaOH solutions. The pH range in this study was selected from (2–7) because at pH ≥ 7, lead(II) ions precipitated as Pb(OH)_2_. While pH_pzc_’s adsorbent charge, at pH above 5 is negative, it is positive at lower pH^13,31,32^. As observed in Fig. 7, upon increasing the pH from 2 to 7, at first, the removal of adsorption on UAS MIL-53(Al) increases from 19 to 86%, meaning that the surface charge of adsorbent is negative. Then pH of 7 causes Pb(II) to hydrolyze in aqueous solution resulting in a slight decrease in adsorption rate. Finally, the optimum pH is obtained at 6. As pH increases, the attraction between Pb^2+^ and the opposing surface increases. As a result, the adsorption rate of lead ions increases upon reducing hydrogen ion concentration which competes with Pb^2+^ ions for the adsorbent sites. The additional parameters to be considered include initial Pb(II) concentration (20 ppm), contact time (60 min), volume of the solution (40 ml), adsorbent dosage of 0.04 g, and temperature of 298 K.

### Effect of adsorbent dosage on Pb(II) adsorption

**Figure 9:**
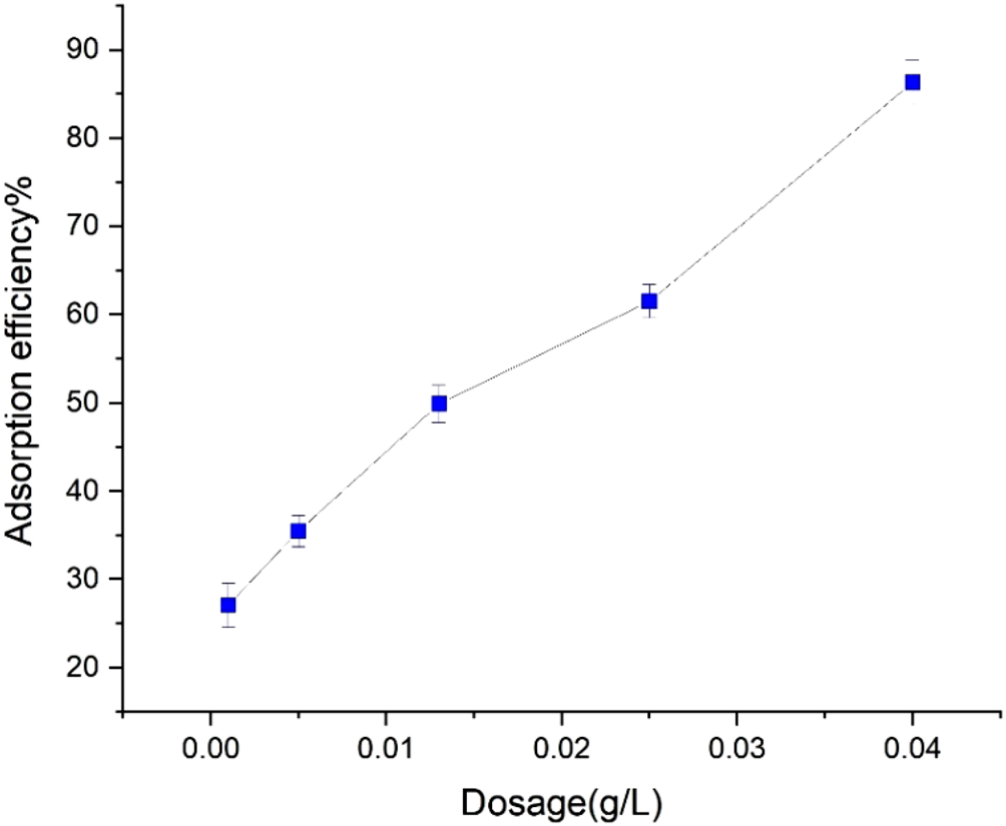
Effect of adsorbent dosage on Pb(II) adsorption (pH of 6, Pb(II) concentration (20 ppm), contact time (60 min), volume of the solution (40 ml), adsorbent dosage range of (0.001–0.04 g), and temperature of 298 K).

The effect of UAS MIL-53(Al) dosage on Pb^+2^ adsorption efficiency was studied in the range of 0.001 to 0.04 g. This study was conducted at a temperature of 298 K and pH of 6 with an initial Pb(II) concentration of 20 ppm for about 60 min. As shown in Fig. 9, the adsorption rate of Pb(II) increases from 27.1 to 86% upon increasing UAS MIL-53(Al) dose, thus increasing the number of available adsorption sites on the UAS MIL-53(Al) surface for binding Pb(II) ions. The significance of the adsorbent dosage indicates its adsorption capacity at a specific initial concentration. It is expected that upon increasing the amount of the adsorbent, the adsorption efficiency will increase due to the increasing amount of adsorbent. Because of the large surface area of the sample, the number of active sites for complexing metal ions increases and the adsorption process accelerates^33^. As the adsorbent reaches its maximum efficiency, the number of active sites on the adsorbent surface corresponds to the number of metal ions available in the solution in the equilibrium adsorption process.

### Effect of initial concentration on Pb(II) adsorption

**Figure 10:**
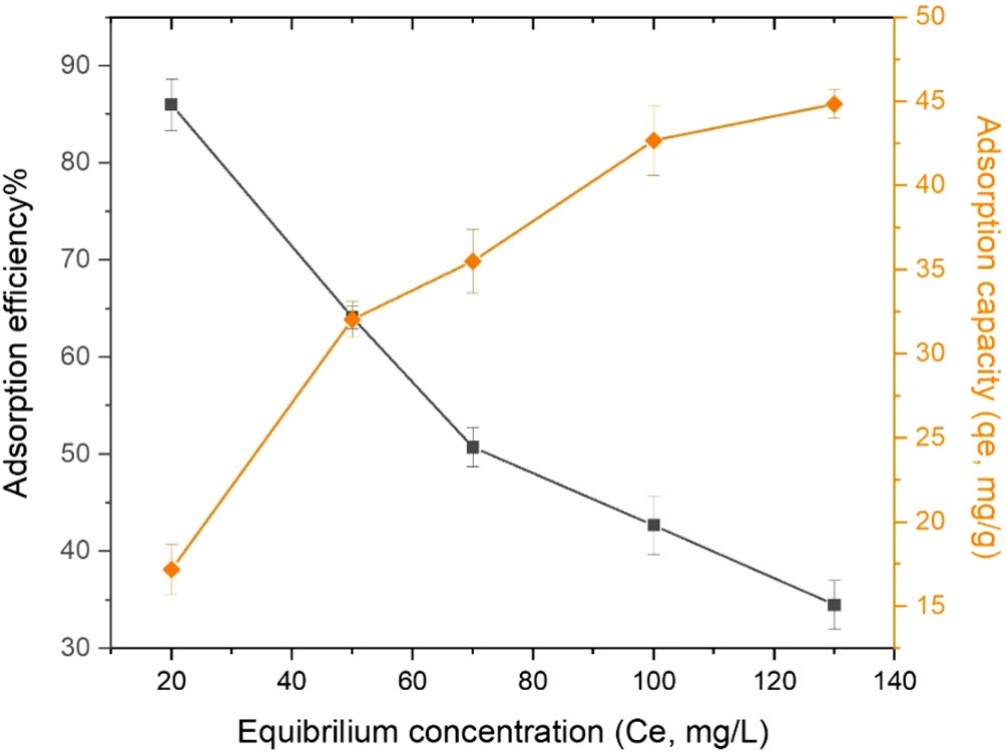
Effect of initial concentration on Pb(II) adsorption (pH of 6, Pb(II) concentration range of (20–130 ppm), contact time (60 min), volume of the solution (40 ml), adsorbent dosage (0.04 g), and temperature of 298 K).

The effect of the initial concentration of UAS MIL-53(Al) on the adsorption efficiency of Pb^+2^ was examined in the range of 20–130 ppm at a temperature of 298 K and pH of 6. As shown in Fig. 10, as the initial concentration of lead(II) ions increases, the removal percentage for this ion decreases from 86 to 34.5%, mainly because the interaction between lead(II) ions and the active adsorbent sites increases at low concentrations, thus leading to a high percentage of adsorption. However, due to the saturation of the adsorbent surface at higher concentrations, more metal ions remain unabsorbed in the solution and the adsorption efficiency decreases upon increasing the concentration. An increase in the concentration of the metal ion would increase the number of collisions between the metal ions and the adsorbent, stimulating the removal of the heavy metal. The number of adsorbed metal ions per adsorbent mass increases in this process. In addition, the adsorption capacity increases as a result of increasing the initial concentrations of metal ions in the solution.

### Effect of contact time on Pb(II) adsorption

**Figure 11:**
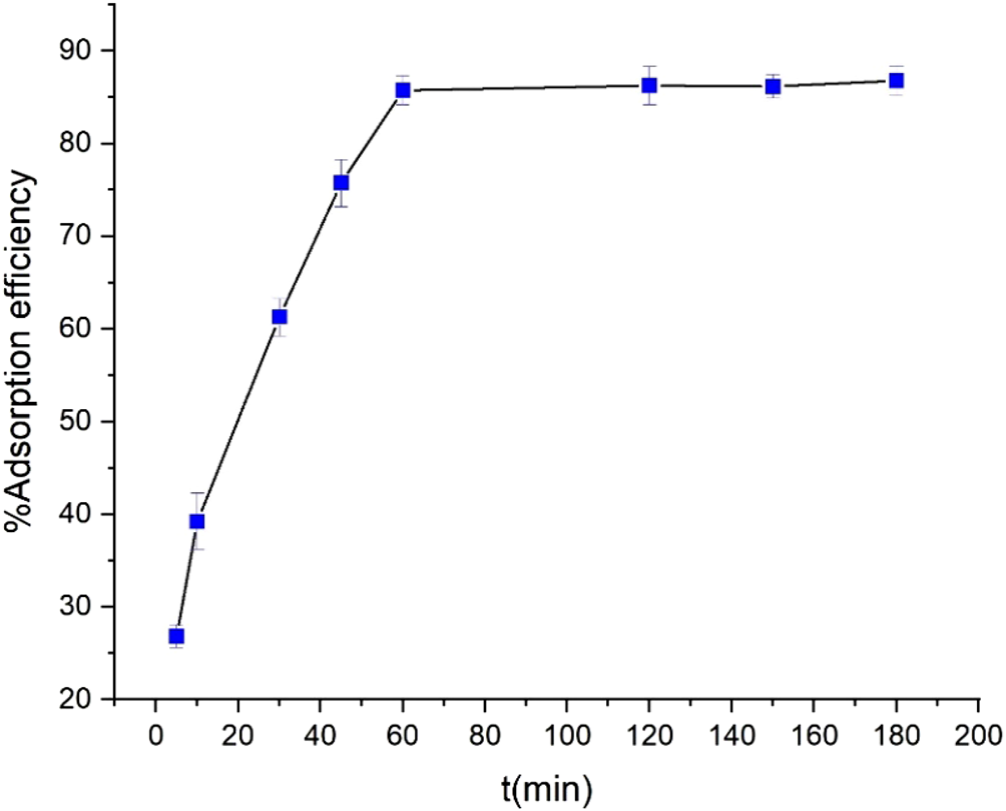
Effect of contact time on Pb(II) adsorption [pH of 6, Pb(II) concentration (20 ppm), contact time range of (5-180 min), volume of the solution (40 ml), adsorbent dosage (0.04 g), and temperature of 298 K)].

The effect of contact time on the adsorption efficiency of lead(II) was investigated using 0.04 g UAS MIL-53 (Al) as an adsorbent in different time durations from 5 to 180 min under some other conditions such as initial Pb(II) concentration of 20 ppm, pH of 6, and temperature of 298 K. As observed in Fig. 11, the adsorption efficiency increases rapidly upon increasing the time in the first 30 min. Finally, by occupying all the active sites on the adsorbent, the adsorption speed decreases considerably and reaches equilibrium in 60 min. Hence, the time limit decreases from 180 to 60 min for all the other experiments. During the first 30 min of the adsorption, the process accelerates owing to the large number of adsorption sites available for metal ions and the adsorbent pores are swiftly filled by the adsorbed ions. Over time, increased adsorption slows down due to such constraints as repulsive forces between the adsorbed metal ions on the surface of UAS MIL-53(Al) and metal ions in the liquid; of note, the adsorption sites remain intact. Moreover, the metal ions are forced to move more profoundly and longer to capture the pores, thus reducing the adsorption rate within minutes. The adsorption process continues until reaching the equilibrium time for adsorption. Equilibrium time is defined as when the adsorption process reaches equilibrium and saturation and the adsorption rate does not change much with time.

### The adsorption kinetics of Pb(II) on UAS MIL-53(Al)

**Figure 12:**
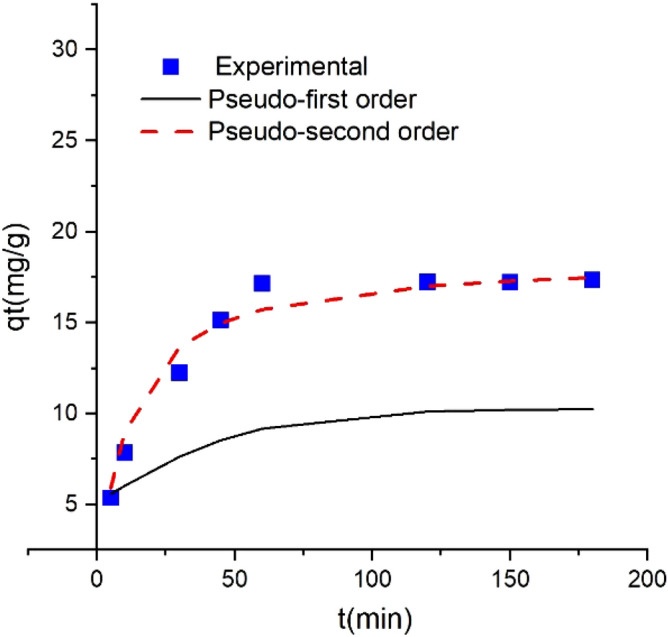
Comparing the theoretical kinetic values with the experimental data.

The kinetic studies were conducted using 0.04 g of UAS MIL-53(Al) as an adsorbent in the condition characterized by different times (5–180 min), pH of 6, and temperature of 298 K using 20 ppm of the initial concentration to remove lead(II) ion from 40 ml solution. To determine the effect of contact time on the adsorption rate and present valuable information about the process mechanism, equilibrium time, and rate control levels, kinetic models were used to test experimental data. The adsorption kinetics of Pb(II) on UAS MIL-53(Al) are shown in Fig. 12. The figure shows the amount of adsorption capacity (mg/g) versus contact time. As discussed before the adsorption capacity swiftly increases in the first 30 min and then the changes were gradually until the equilibrium was reached after 60 min. In order to analyze the data and evaluate the adsorption quality different kinetic models including pseudo-first-order and pseudo-second-order as well as intra-particle diffusion kinetic models are employed^34^.

### Pseudo first-order kinetic model

**Figure 13:**
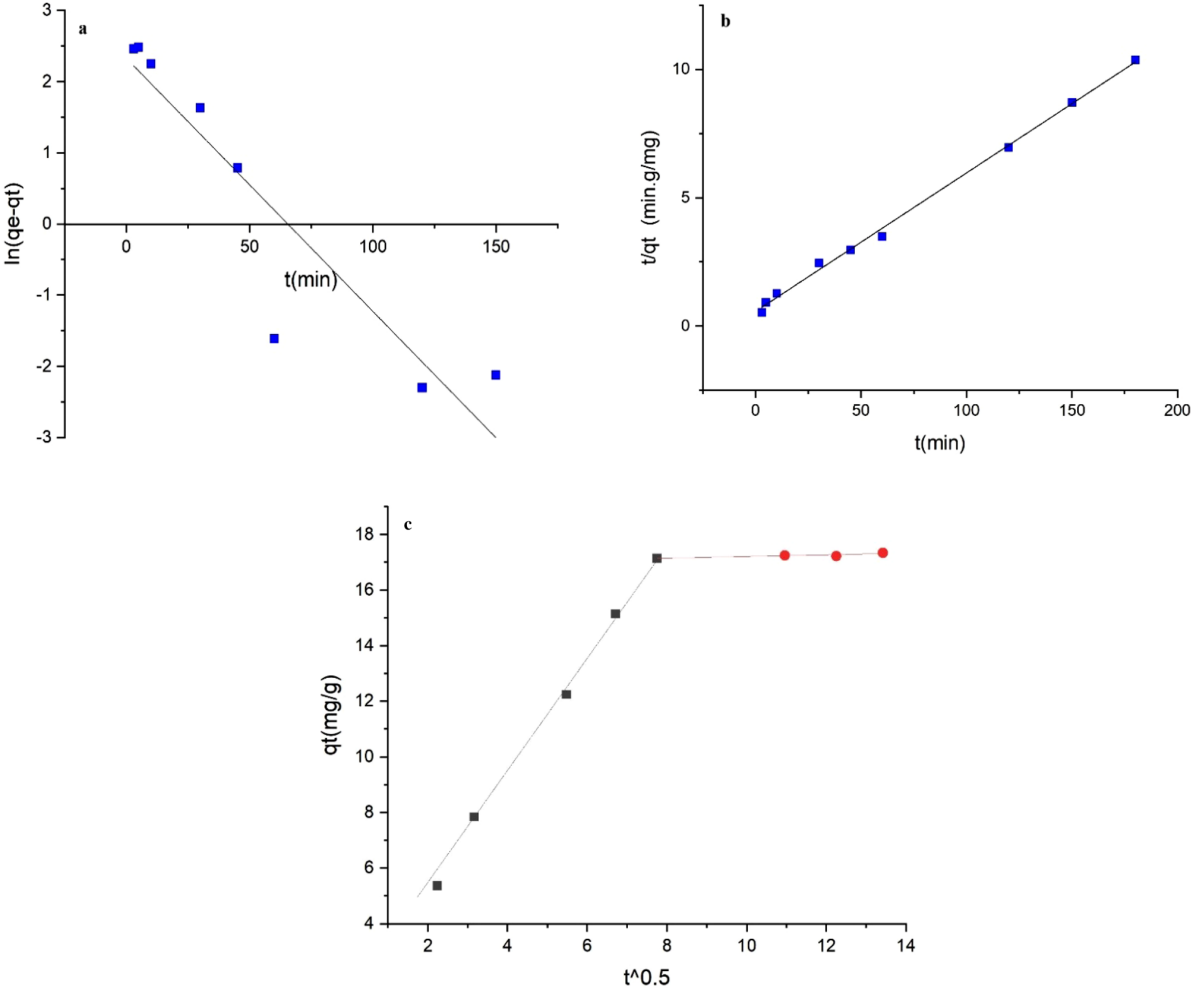
(**a**) Pseudo first-order kinetic, (**b**) pseudo second-order kinetic, (**c**) intr-aparticle diffusion model [(pH of 6, Pb(II) concentration (20 ppm), contact time range of (5–180 min), volume of the solution (40 ml), adsorbent dosage (0.04 g), and temperature range of (298 K)].

The Pseudo first-order rate is presented in^35^ Eq. (3).

3 $$\mathrm{ln}({q}_{e}-{q}_{t})=ln{q}_{e}-{k}_{1}t$$

in which q_e_(mg/g) is the amount adsorbed equilibrium, q_t_(mg/g) the amount adsorbed at any time, and K_1_ (min^−1^) a pseudo-first-order rate constant. The k_1_ and ln q_e_ of the pseudo-first-order model can be obtained from the slope and intercept of ln (q_e_ − q_t_) vs. time plot (Fig. 13; Table 2). The theoretical plot of qt vs. time using pseudo first-order is depicted in Fig. 12, indicating that the theoretical values of q_t_ do not match the experimental data. Therefore, this kinetic model does not adequately describe the adsorption process. The theoretical data's R^2^ and q_e_ values are shown in Table 3.

**Table 2:** Kinetic- model parameters for the adsorption of Pb(II) on UAS MIL53(Al).

Models	Parameters	Data
Pseudo first-order	q_e_	10.26
	K_1_	0.0356
	R^2^	0.8547
Pseudo second-order	q_e_	18.518
	K_2_	0.00508
	R^2^	0.9975

**Table 3:** Intra-particle diffusion model parameters for the adsorption of Pb(II) on UAS MIL53(Al).

	First stage			Second stage		
	C	K_i_	R^2^	C	K_i_	R^2^
UAS MIL-53(Al)	1.472	2.0146	0.993	16.909	0.0303	0.811

### Pseudo-second order kinetic model

The pseudo-second-order rate is determined using^34^ Eq. (4).

4 $$\frac{t}{{q}_{t}}=\frac{1}{{ {q}_{e}^{2}K}_{2}}+\frac{t}{{q}_{e}}$$

in which K_2_(g/g min) is a pseudo-second-order constant. The k_2_ and q_e_ of the pseudo-second-order model can be obtained by plotting the t/q_t_ vs. time plot (Fig. 13; Table 2). The values of 1/q_e_ and 1/k_2_ q_e_^2^ can be obtained from the slope and intercept, respectively. The theoretical plot of q_t_ vs. time using pseudo-second-order is shown by dash line in Fig. 12. The consistency between the experimental and theoretical data regarding pseudo-second-order kinetic model can be confirmed from Fig. 12. The R^2^ and q_e_ values of pseudo-second-order are given in Table 3; The q_e_ values calculated from pseudo-second-order kinetic (18.51 mg/g) is more consistent with the experimental q_e_ value found at 17.35 mg/g than that calculated from the pseudo-first-order model, showing that the pseudo-second-order kinetic model described the adsorption process better.

### Intra-particle diffusion kinetic model

Equation (5) defines the intra-particle diffusion rate to detect the diffusion mechanism and rate control steps of the adsorption process^36^.

5 $${q}_{t}={K}_{i}{t}^{0.5 }+c$$

in which K_i_ is the intra particle diffusion rate constant (mg/(g.min^0.5^)) and C(mg/g) is the intercept value (Table 3; Fig. 13). The theoretical plot of q_t_ versus time using intra particle diffusion kinetic model is shown by dash line in Fig. 12. Commonly, the overall adsorption process may be controlled by several steps, e.g., adsorbent mass transfer occurs across the boundary layer (film diffusion), pore diffusion, surface diffusion, and intra particle diffusion. The slowest stage is used to manage the overall rate of the adsorption process^37^. Intra particulate diffusion predominantly contributes to the rate-limiting step if the correlation diagram of adsorbed ions (q_t_) versus (t^0.5^) gives a straight line through the origin. If not, the plot may present a multi-linearity demonstrating that multiple steps, such as boundary layer diffusion or other processes. Moreover, the intercept value (C) represents the boundary layer thickness, and a higher C value specifies a thicker boundary layer. As illustrated in Fig. 13, adsorption occurs in two stages, the first and final stages. It is assumed that the initial stage with a sharper slope indicates that the adsorption follows diffusions with the particles or mass transfer. The second section, where slope k is close to zero, is the gradual adsorption stage with controlling intra-particle diffusion^37,38^. The R^2^, C, and K_i_ values are obtained from the second stage (intra particle diffusion), and the results are given in Table 2. Figure 13 indicates that intra particle diffusion is not the only rate-limiting step since the straight lines do not pass from the origin^39^.

### Adsorption isotherms

The adsorption isotherm models proposed by Langmuir^40^, Freundlich^40^, Temkin^40^ were applied at a temperature of 298 K with different lead(II) concentrations of 20, 50, 70, 100, and 130 ppm, 0.04 g of UAS MIL-53(Al), and pH of 6 for 60 min to investigate the interaction between equilibrium concentration data and lead(II) ion adsorption.

### Langmuir isotherm

**Figure 14:**
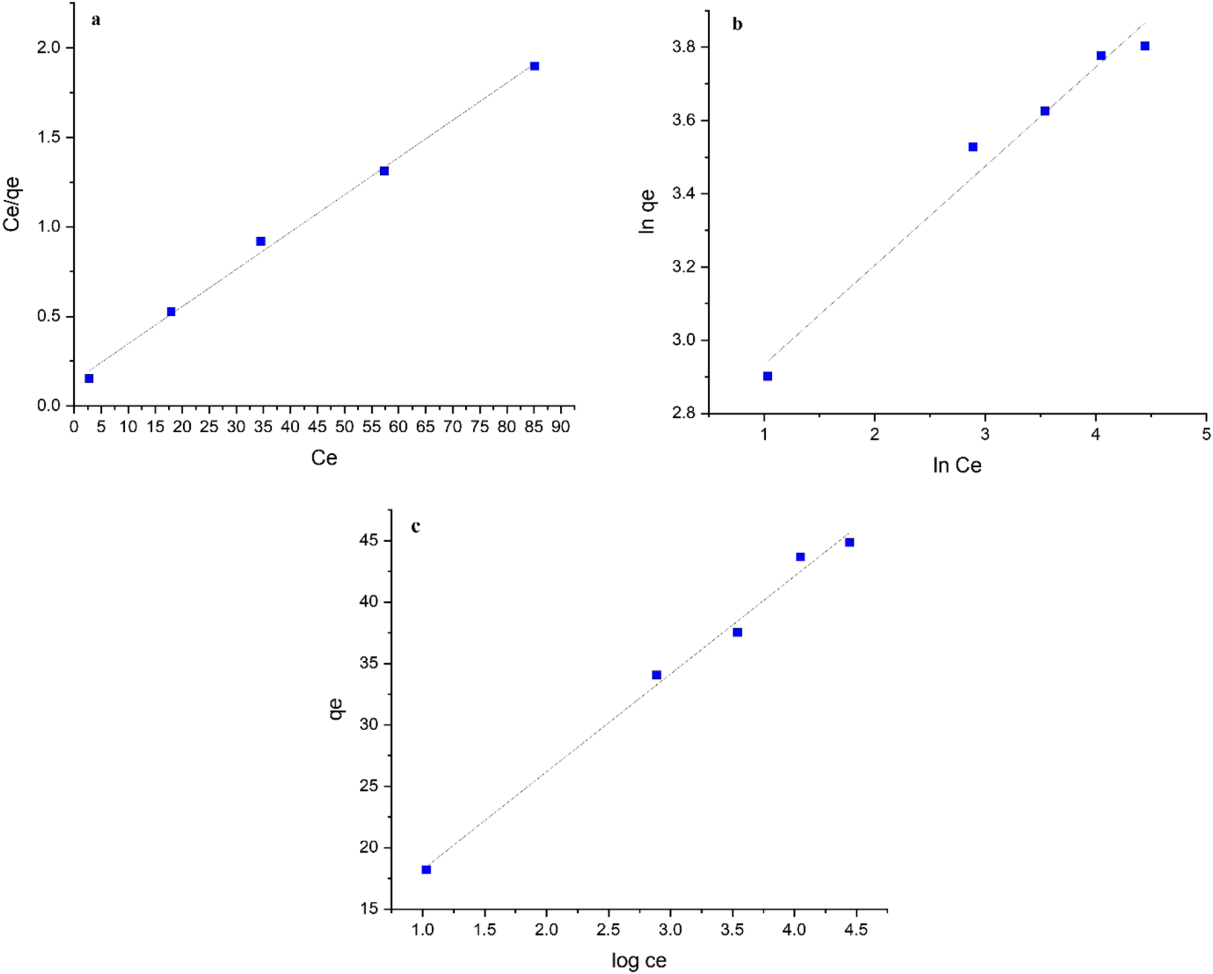
(**a**) the Langmuir, (**b**) the Freundlich, and (**c**) the Temkin isotherms ((pH of 6, Pb(II) concentration range of (20–130 ppm), contact time (60 min), volume of the solution (40 ml), adsorbent dosage of (0.04 g), and temperature of (298 K)).

This isotherm is employed to adsorb a dynamic equilibrium surface on perfectly homogeneous surfaces, assuming a monolayer coating onto the adsorbent surface area. In addition, due to the occupation of the adsorption sites, the adsorbed molecules do not interact with each other. The parameters of the Langmuir isotherm can be calculated through plotting its linear diagram in Eq. (6):

6 $$\frac{{c}_{e}}{{q}_{e}}={c}_{e}\frac{1}{{q}_{m}}+\frac{1}{{{k}_{l}q}_{m}}$$

where C_e_ (mg/L) is the metal ion concentration, q_e_(mg/g) the number of metal ions adsorbed in the equilibrium phase, q_m_ the maximum monolayer adsorption (mg/g), and k_l_ (L /mg) the Langmuir constant related to the adsorption energy. The values for the Langmuir constants k_l_ and q_m_ were achieved from slope and intercept of C_e_ versus C_e_/q_e_ plot given in Table 4 and Fig. 14. In case R^2^ is more than 0.99, the surface assimilation of the method follows the Langmuir isotherm. The dimensionless equilibrium parameter R_L_ is defined to explain the type of isotherm (if R_L_ = 0, irreversible; R_L_ > 1, unfavorable; 0 < R_L_ < 1, favorable; and R_L_ = 1, linear) in the adsorption process obtained from^41^ Eq. (7).

**Table 4:** Isotherm parameters values of Langmuir, Freundlich, Temkin models for the adsorption of Pb(ll) on UAS MIL-53(Al).

Isotherm models	Parameters	Adsorption of lead(II) on UAS MIL-53(Al)
Langmuir	Temp q_m_ (mg/g) K_L_ (l/mg) R^2^ R_L_	298 K 48.076 0.149 0.9965 0.049
Freundlich	Temp K_f_ n R^2^	298 K 14.34 3.6941 0.9763
Temkin	Temp K_T_(L/g) B_T_ (J/mol) R^2^	298 K 3.9226 9.7037 0.9712

7 $${R}_{l}=\frac{1}{1+{K}_{l }{C}_{0}}$$

where C_0_ is the initial concentration (mg/L). When the value of R^2^ is 0.9965, the Langmuir isotherm fits the adsorption data and since 0 < R_L_ < 1, the type of Langmuir isotherm is favorable. The maximum monolayer adsorption (q_m_) can be achieved by linear Langmuir isotherm that is estimated to be 48.076 mg/g near the values of experimental maximum adsorption capacity (44.85 mg/g).

### Freundlich isotherm

Freundlich equilibrium isotherm model can illustrate the concept of heterogeneous surfaces and assume adsorption on the multilayer surfaces. The parameters of this model (k_f_ and n) can be calculated from the intercept and slope of ln q_e_ versus ln C_e_ diagram, respectively, using Eq. (8).

8 $$\mathrm{ln}{q}_{e}=\mathrm{ln}{K}_{f}+ \frac{1}{n} (\mathrm{ln}{C}_{e})$$

where K_f_ is an adsorption isotherm constant that signifies an approximate adsorption capacity and n is the Freundlich equilibrium constant related to the adsorption intensity. If the value of n ranges from one to ten, the adsorption is assumed favorable. In the case of n = 3.6941, the adsorption process is favorable. The values of Freundlich constants are given in Table 4 and Fig. 14.

### Temkin isotherm

Temkin isotherm is elaborated in Eqs. (9) and (10). Through this model, the effect of the indirect interaction of adsorbent on the heat reduction of the adsorption can be clarified^39^.

9 $${q}_{e}={B}_{T}ln{A}_{T}+{B}_{T}ln{C}_{e}$$

10 $${q}_{e}=\frac{RT}{{B}_{T}}ln{K}_{T}+\frac{RT}{{B}_{T}}ln{C}_{e}$$

B_T_ (J/mol) constant denotes the heat of the adsorption coverage, and K_T_ (L/g) is a constant of the binding energy, indicating the significant connection between the adsorbate and adsorbent^39^. Moreover, the Temkin isotherm (K_T_ and B_T_) constants can be measured by plotting ln q_e_ versus ln C_e_ diagram from the intercept and slope, respectively. The isotherm model plots are given in Fig. 14, and all the constant values are summarized in Table 4.

### Comparison of different adsorbents performances in Pb(II) removal

Table 5 compares the lead (II) removal data using various adsorbents. It was found that a significantly small amount of UAS MIL-53(Al) had a moderately better adsorption ability than other samples in a short time. The difference in adsorption rate and capacity of the listed adsorbents lies in different conditions of the adsorption performance, such as temperature, the mass of adsorbent, contact time, the volume of the solution, and difference in surface areas and functional groups. Principally, UAS MIL-53(Al) managed to have a very high adsorption efficiency of 97.63% in comparison to some other adsorbents at pH 6, due to its hierarchical structure and vaster surface area. Two key factors influence the adsorption performance the most, including specific surface area and adsorbent-adsorbate interactions, which can be optimized by doing some pre/post-treatments. As shown in Table 1, UAS MIL-53(Al) has a greater surface area comparing the conventional one CS MIL-53(Al), 1538.6 and 1274.3 (m^2^/g), respectively. Although the nature of both adsorbents and so their interactions to the adsorbate is the same, the one with the more specific surface area has a greater adsorption performance of 97.63%, which shows the importance of the surface area in providing the more accessible active sites for surface adsorption. Besides, comparing with MIL possessing functional groups^13^, although the adsorbent has a low surface area it shows the great adsorption performance because of adding functional groups which resulted in more adsorbent- adsorbate interactions.

**Table 5:** Comparison of adsorption parameter for Pb(II) on different adsorbents.

Adsorbent	Ci (ppm)	Contact time (min)	Mass of adsorbent	pH	q (mg/g)	T(K)	Adsorption efficiency %	Refs
UAS MIL-53(Al)	20	60	0.04 g	6	19.526	318	97.63	This study
CS MIL-53(Al)	20	60	0.04 g	6	10.04	318	50.2	This study
SAPO-5	60	180	0.12 g	5–6	17.29	–	80.5	^34^
AIPO-5	60	180	0.12 g	5–6	19.9	–	84.5	^34^
AMCA-MIL53(Al)	400	120	0.075 g	5.8	390	318	92	^13^
Magnetic chrysotile Nanotubes	21.4	–	0.57 g/l	5	27.64	298	–	^42^
Mesoporous silica	828	20	0.05 g	6	84.49	–	51	^43^
ED-MIL-101(Cr)	50	30	0.02 g	6	81.5	298	–	^44^

### Regeneration of adsorbent

**Figure 15:**
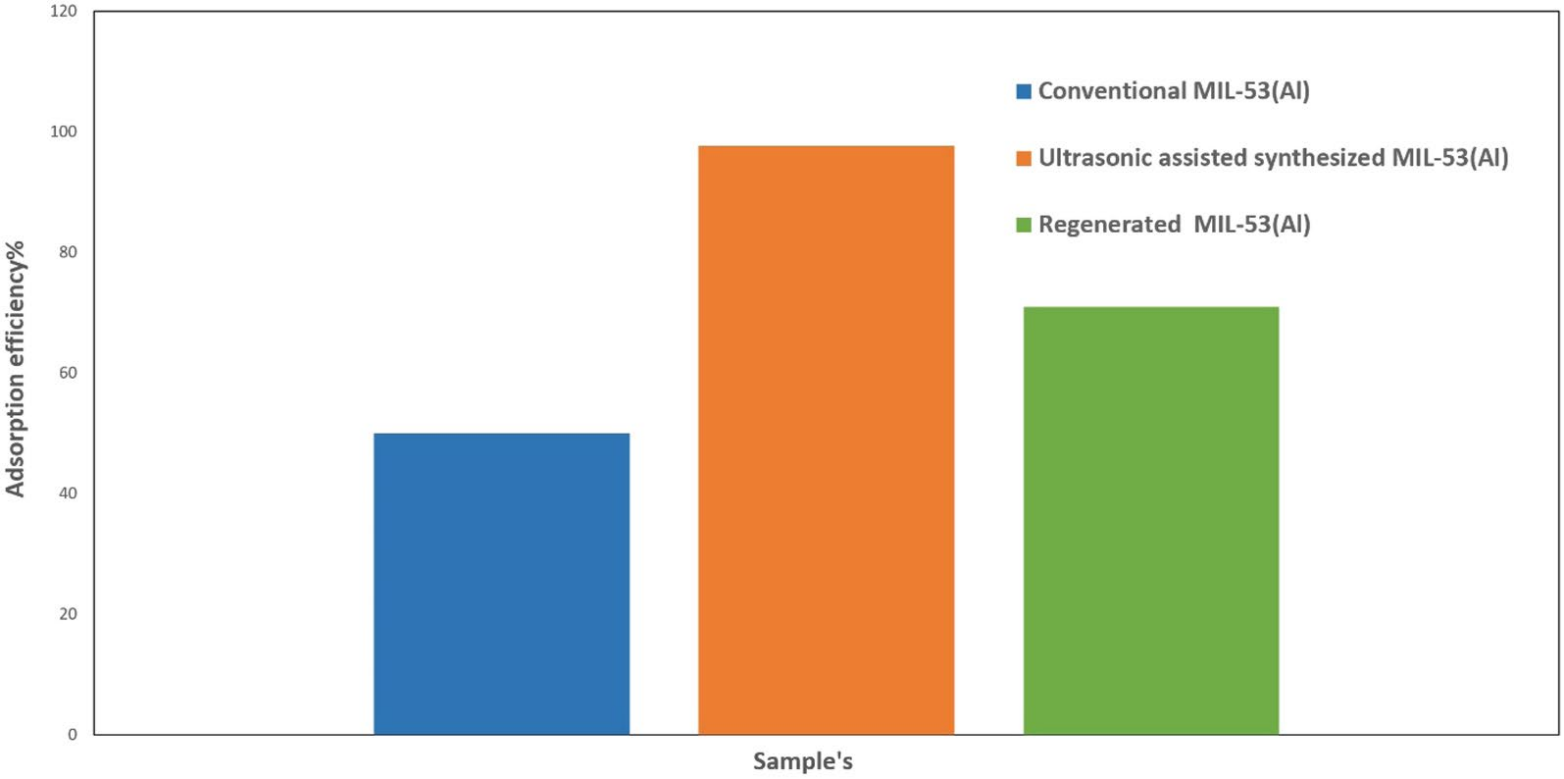
Comparison of adsorption parameter for Pb(II) on conventional and ultrasonic assisted synthesized and 1 cycle regeneration of adsorbent.

The regeneration efficiency of UAS MIL-53(Al) as an adsorbent was evaluated with various elements such as H_2_SO_4_, and HCl. HCL 0.1 M as a strong acid was discovered to be the best desorption of Pb^+2^ ion to wash the adsorbent with it, rewash it with distilled water, and dry and check its recyclability adsorbent. 0.1 M HCl was used to wash up the Pb(II) for one cycle. The Comparison of adsorption for Pb(II) on Conversion and Ultrasonic synthesis and regeneration of adsorbent in one cycle has shown in Fig. 15.

### Mechanism of adsorption

**Figure 16:**
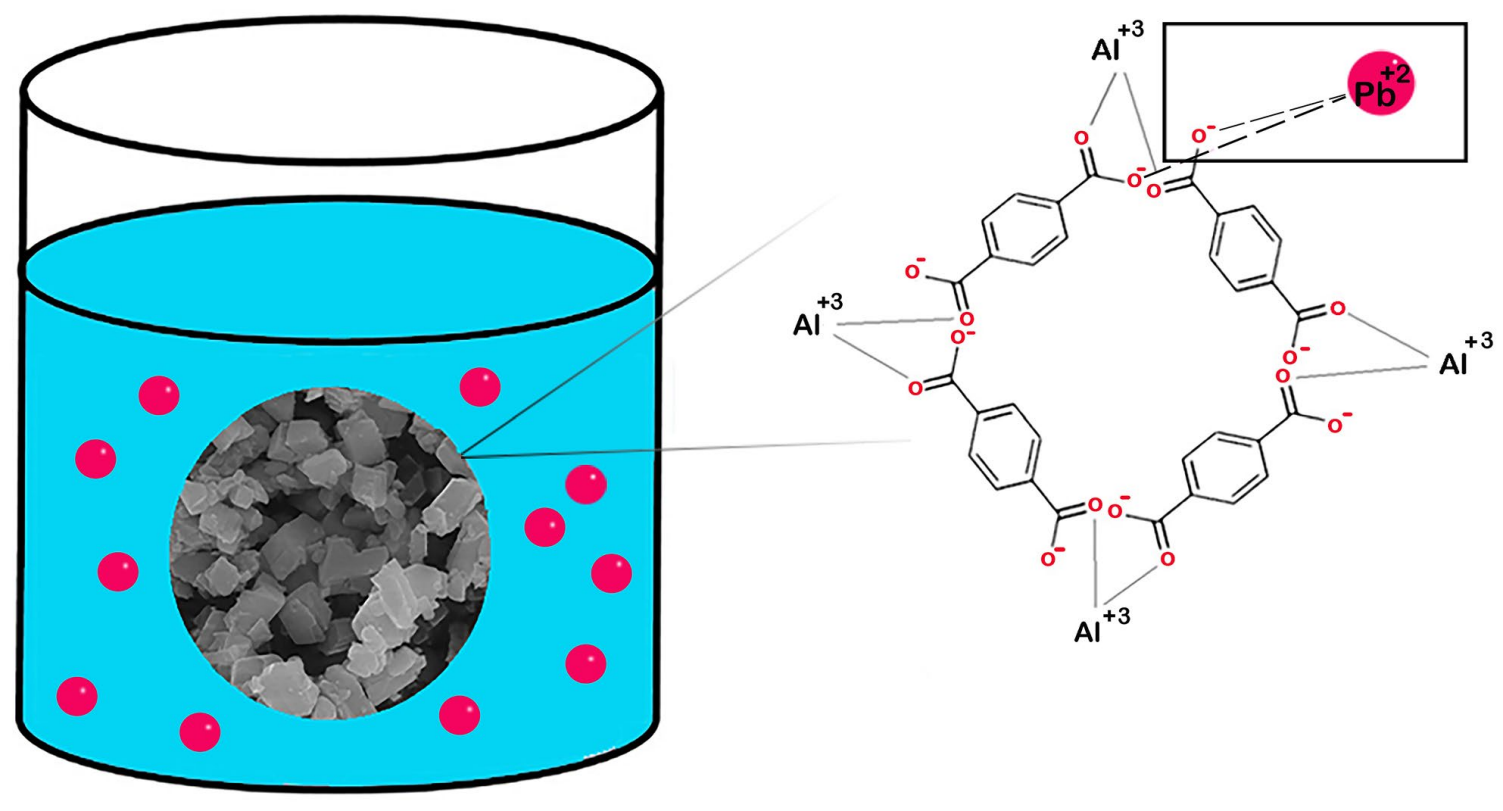
The schematic representation of adsorption mechanism of Pb^+2^ on UAS MIL-53(Al).

According to the pseudo-second-order kinetic model, the adsorption mechanism is chemical. Figure 16 proposes the adsorption mechanism schematically. It is observed that MIL-53(Al) has oxygen atoms with free negative charges, which attracted positively charged Pb^+2^. Thus, chemo sorption occurred as a result of an electrostatic attraction between electronegative oxygen atoms and electropositive Pb^+2^.

## Conclusion

In this study, ultrasonic assisted synthesis (UAS) of MIL-53(Al) and its adsorption performance in removing of lead (II) ions from aqueous solution were examined. Comparing the conventional synthesis, the sonochemical synthesis leads to shorten the synthesis time from 3 days to 1 day, resulting in energy consumption reduction, faster reaction rate. The usage of ultrasound in MIL-53(Al) synthesis additionally creates the hierarchical structure, increases the relative crystallinity and specific surface area of the sample up to 1538.6 m^2^/g. The optimal conditions for lead(II) adsorption were achieved using 0.04 g of UAS MIL-53(Al) adsorbent, 60 min of contact time as equilibrium time, an initial concentration of 20 mg/L, and maximum pH of 6. UAS MIL-53(Al) confirmed the adsorption efficiency of 97.63% at 315 K. The kinetic data followed a pseudo-second-order rate equation where lead(II) ions were adsorbed onto various sites at different points. Also, the equilibrium data fit the Langmuir isotherm model. In summary, it is expected to use UAS MIL-53(Al) as a promising adsorbent for lead(II) removal from aqueous solutions.
